# Plastics as disruptors of feeding, digestive physiology, metabolism, and growth in fish and other aquatic ectothermic vertebrates

**DOI:** 10.3389/fendo.2026.1873239

**Published:** 2026-06-26

**Authors:** Thanushanthahi Loganathan, Helene Volkoff

**Affiliations:** Department of Biology, Memorial University of Newfoundland, St. John’s, NL, Canada

**Keywords:** appetite regulation, aquatic ectotherms, energy metabolism, gut physiology, plastics

## Abstract

Plastics, particularly microplastics (MPs) and nanoplastics (NPs), are widespread contaminants in aquatic ecosystems that affect key physiological processes related to feeding, digestion, metabolism, and growth in aquatic ectotherms, particularly fish. Increasing evidence indicates that plastic exposure disrupts energy balance by reducing food intake through false satiety, gastrointestinal obstruction, and behavioral alterations, while also impairing digestive efficiency, nutrient absorption, and metabolic regulation. MPs and NPs can interfere with endocrine signaling pathways involved in appetite regulation. They may also disrupt the thyroid axis, a key regulator of metabolism and energy expenditure, and the growth hormone/insulin-like growth factor axis, which controls somatic growth and nutrient partitioning. These endocrine disturbances are often accompanied by oxidative stress, impaired hepatic function and gastrointestinal integrity, ultimately affecting growth performance, energy allocation, and overall fitness. While these effects are best documented in fish, amphibians show similar but less well-characterized responses, whereas evidence in reptiles remains limited and largely observational. The impacts of plastics are further modulated by environmental conditions associated with climate change. Factors such as temperature and salinity can influence the uptake, bioavailability, and toxicity of MPs and NPs, often exacerbating their effects on feeding behavior, metabolic performance, and endocrine function. In addition, climate-driven processes -including warming, extreme weather events, and changes in ocean circulation - can alter the breakdown, transport, and distribution of plastics, potentially increasing exposure risks. However, high heterogeneity in experimental protocols, such as differences in plastic characteristics, exposure concentrations and durations, species, and life stages, limits direct comparison across studies. This review synthesizes current knowledge on the effects of plastic exposure on feeding, digestion, metabolism, and growth in aquatic ectotherms, with a particular focus on fish, while integrating available evidence from amphibians and reptiles. It also highlights underlying mechanisms and the influence of environmental conditions, with implications for fish health, aquaculture productivity, and ecosystem functioning in increasingly polluted aquatic environments.

## 1 Plastics in aquatic environments

Plastics are found in both marine and freshwater ecosystems (1–4). In freshwater systems, including rivers, lakes, and reservoirs, contamination originates mainly from land-based human activities, entering ecosystems as both large plastics (macroplastics) and microplastics (5, 6). Major sources of plastic pollution include wastewater (water contaminated by domestic, commercial, and industrial use) and urban runoff (precipitation that collects surface pollutants as it flows over impermeable surfaces). These pathways transport plastics (including microfibers from textiles, microbeads from personal care products, and road-derived plastics) into aquatic ecosystems, primarily via wastewater treatment plant effluents, which incompletely remove plastics, and sewer overflows. Additionally, stormwater drains act as direct sources for larger debris and road-derived microplastics, such as tire wear particles, road markings, and consumer packaging, often discharging them directly into streams with minimal or no filtration. Additional contributions arise from industrial activities, as well as debris associated with agricultural plastics and fisheries and recreational uses (e.g., packaging materials, greenhouse coverings, fishing lines, and nets) (7, 8).

In addition to urban wastewater and runoff, atmospheric deposition and long-distance transport via wind or precipitation can deliver microplastics to remote lakes and headwaters, even in areas remote from direct urban sources (2, 6, 9). On a global scale, it is estimated that 70–80% of marine plastic originate from land-based sources, but ocean−based activities including fisheries, aquaculture, shipping, offshore industries and tourism are also significant contributors to the total plastic load (10, 11).

Climate change can influence the distribution and transport of plastics in aquatic systems through environmental processes (12, 13). Extreme weather events, such as storms and flooding, increase surface runoff and facilitate the transfer of plastics from terrestrial environments into rivers and oceans. In marine systems, alterations in ocean circulation and stratification can further redistribute plastics, modifying their spatial accumulation patterns. Additionally, sea ice and glaciers may act as temporary sinks, trapping MPs and releasing them during melting, thereby contributing to inputs into aquatic environments.

## 2 Classification and concentrations of plastics in aquatic environments

Plastics in aquatic environments are usually classified based on three main criteria: size, origin, and polymer type. Size-based classification is the most widely used, although size thresholds vary among studies.

Plastics are generally classified by size into microplastics (MP, <5 mm), mesoplastics (~5 -20/25 mm), macroplastics (~20/25–100 mm), and megaplastics (>25–100 mm), with macro- and megaplastics visible to the naked eye. The term microplastics is widely used to describe small plastic particles, even though no clearly lower size limit has been established. Over time, MPs can further fragment into nanoplastics (NP, ~1–1000 nm), which are recognized as environmentally relevant but remain difficult to detect and quantify (2, 14, 15). MPs are often categorized as primary MPs, which are manufactured at small sizes (e.g., cosmetic microbeads, industrial pellets, pre-production nurdles) or secondary MPs, which result from the fragmentation of larger plastic items (e.g, bags, bottles, fishing gear and textiles) through processes such as ultraviolet (UV) radiation, mechanical abrasion and biodegradation (14, 16, 17).

Across both marine and freshwater systems, the most frequently detected plastic polymers are polyethylene (PE), polypropylene (PP), polystyrene (PS), and polyethylene terephthalate (PET), with cellulose acetate, polyvinyl chloride (PVC), polyamide (PA/nylon), polyesters and acrylics also commonly reported (14, 18–20). Polymer composition influences the distribution of plastic debris in aquatic systems, in particular their vertical distribution in the water column. Low density polymers such as PE and PP, are less dense than water and tend to float whereas higher density polymers such as PET, PVC, polyesters and PA, tend to sink and accumulate in sediments (15, 17).

Climate change alters key environmental conditions in aquatic systems, including temperature, salinity, and UV exposure (13). Increasing global temperatures warm water bodies, while shifts in precipitation, evaporation, and freshwater inputs modify salinity. Changes in atmospheric conditions and ice cover can also influence UV radiation penetration. Together, these factors can affect the generation, transport, and fate of MPs/NPs by altering degradation rates and particle properties (13, 21).

The abundance and distribution of plastics vary widely across studies, depending on region, proximity to anthropogenic sources, hydrodynamics, polymer type, particle size, and sampling methodology (22, 23). MPs are the most extensively studied, with concentrations in marine systems ranging from as low as 10–^2^ particles/m3 up to over 16000 particles/m^3^. levels are typically highest in nearshore areas and estuaries and lowest in the open sea (22, 24, 25). In freshwater systems, reported concentrations range from 10^-2^ to 10^8^ particles/m³, with higher levels in rivers and lakes influenced by agricultural, urban, or industrial activities and lower levels in remote or minimally impacted waters (26–31). Sediment concentrations can reach thousands of particles per kilogram (24, 29). Data for small plastics, particularly NPs, remain limited due to the lack of reliable quantitative detection methods (22, 32, 33). NPs have only recently been quantified in marine systems, with concentrations of 1.5–32 mg/m³ reported in the North Atlantic (34). In freshwater, environmental levels are still poorly characterized but have been reported to range from 300 μg/m^3^ to 0.5 g/m³ (35). Mesoplastics are less frequently measured, although available studies indicate they can occur at significant levels alongside MPs (36–38).

Direct comparison among studies is complicated by the lack of standardization in reporting units and methodologies. Plastic concentrations are reported using different metrics, including particle-based units (e.g., particles/L or/m^3^), surface-area-based units (e.g., particles/Km^2^ in net-tow studies) and mass-based units (e.g., mg/L, ppm, or g/L). In addition, key particle characteristics such as size, density, and shape are often not reported. To facilitate rough comparison with studies reporting mass-based exposure concentrations, approximate mass concentrations can be derived by assuming an average particle diameter (~70μm for MPs and ~30nm for NPs) and a polymer density of 1 g/cm^3^. based on these assumptions, reported concentrations of MPs correspond approximately to 2x10^-6^ to 3μg/L in marine systems and 2x10^-6^μg/L to 20 mg/L in freshwater. For NPs, estimated concentrations would range from 1.5 to 30μg/L in seawater and 0.3 to500μg/L in freshwater systems. However, these estimates should be interpreted cautiously given the simplifying assumptions involved.

Methodological differences further contribute to variability among studies. For example, net tows and trawls typically target larger size particles and may miss smaller particles, whereas pump filtration and spectroscopic identification can detect smaller MPs and often yield higher reported concentrations than those obtained using net-based sampling (22, 28).Variations in mesh size, sampling depth, and analytical detection further influence reported values. Together, these differences contribute to the wide variability in reported concentrations and complicate direct comparisons among studies, as well as comparisons between field measurements and laboratory exposure levels.

## 3 Exposure pathways in aquatic ectothermic vertebrates

Plastic pollution, particularly in the form of MPs and NPs represent a widespread threat to aquatic ecosystems (39). Numerous studies demonstrate that plastics are ingested by a wide range of aquatic ectothermic vertebrates.

Aquatic ectothermic vertebrates, including fish, amphibians, and to a lesser extent, reptiles, are particularly susceptible to plastic pollution due to their continuous exposure to the surrounding environment and their ectothermic physiology, which is directly influenced by environmental factors. For example, temperature-mediated changes in metabolic rate and activity can in turn affect feeding behavior and the likelihood of plastic ingestion. Understanding exposure to plastics in aquatic ectotherms can therefore provide important insights into their biological effects.

Exposure occurs through multiple pathways, with direct oral ingestion from the water column or contaminated sediments being the dominant route, accounting for more than 70% of MP accumulation and primarily affecting the gastrointestinal tract (40–42). Plastics may be mistaken for prey items or ingested incidentally during feeding or filter-feeding (40, 43). Additional exposure routes include gill retention, which occurs passive gill retention during respiration, and, to a lesser extent, skin adhesion, which may cause physical damage and inflammatory responses, particularly in scaleless fish (40, 43). NPs are of particular concern due to their small size, which allows them to cross biological membrane and accumulate within cells and organelles, and persist long enough to induce oxidative and genotoxic effects (44–48). Consistent with this, small MPs and NPs have been detected beyond the gastrointestinal tract, suggesting that MP/NPs can cross the intestinal barrier and travel via the bloodstream to organs such as the liver, brain and gonads [fish (40, 49–51); amphibians (52); reptiles (53)].

Exposure and accumulation are modulated by species-specific traits, habitat, feeding strategy, and life stage (54). For example, early life stages of fish, including embryos, larvae, and juveniles, appear particularly susceptible to MP pollution (55). Feeding habits also influence both the quantity and characteristics of ingested plastics, although this relationship remains unclear. Some studies report higher ingestion rates in omnivorous and herbivorous fish (56–62), possibly because omnivores exploit a wider range of food sources, increasing their likelihood of consuming MP-contaminated items, while herbivores may be exposed to plastics adhered to algae and plant surfaces or accumulated in detritus and sediments during grazing (61). In contrast, other studies report higher plastic ingestion in carnivores (63, 64).

Trophic level may also influence plastic accumulation, but findings are inconsistent. Some studies report higher MP concentrations in fish species at higher trophic levels, consistent with trophic transfer through prey consumption (63, 65). However, other studies show greater MP accumulation in lower-trophic-level fish, particularly of smaller particles. This pattern may reflect direct ingestion from water or sediments, or consumption of contaminated small prey such as zooplankton. Independently of trophic position, habitat and feeding mode also influence MP exposure: for example, benthic species, that feed near or on sediments, often show higher MP loads than pelagic species, likely due to greater exposure to sediment-associated MPs (62, 66).

Several studies show that behavioral phenotype and intra-individual variations in behavior (“animal personality”) affect MP ingestion rates. For example, bold zebrafish (*Danio rerio*) capture and ingest MPs more frequently than shy individuals, indicating personality-linked exposure risk (67, 68). In juvenile anemonefish (*Amphiprion ocellaris*), more active individuals ingest more MP particles than less active individuals when exposed to the same particle concentration (69). In Western mosquitofish (*Gambusia affinis*), dominant fish are the first to approach food and have higher feeding activity, including when foraging for MPs, than subordinate fish, suggesting that dominant fish may be more exposed to MPs (70). Similarly, in European minnow (*Phoxinus phoxinus*), dominant individuals consistently capture and ingest more MPs than subordinates (71).

## 4 Effects of MP/NP in food intake and feeding behavior

An increasing number of studies have shown that MPs can impair food intake and feeding behavior across a wide range of fish species, primarily through false satiety, physical blockage of the gut, or behavioral disruption.

### 4.1 Fish

Overall, findings suggest that plastic exposure disrupts feeding behavior in fish, predominantly reducing food intake, although responses seem to be dependent on species, particle characteristics such as size and concentration, as well as life stage. MP exposure decreases feeding in jacopever (*Sebastes schlegelii*) (PS-MPs 10^6^ microspheres/L for 14 days) (72), rainbow trout (*Oncorhynchus mykiss*) (PS-MPs 5% in feed for 6 weeks) (73), pond loach (*Misgurnus anguillicaudatus*) (PE-MPs 1 mg/L for 14 days) (74), goldfish (*Carassius auratus*) (1000 microplastic fibers/L for 45 days; PS-MPS 0.69 mg/L for 28 days) (75, 76), catla (*Catla catla*) (PS-MPs 2.5% in feed for 90 days) (77), walking catfish (*Clarias batrachus*) (MPs 5% in feed for 60 days) (78) and Nile tilapia (*Oreochromis niloticus*) (MPs 8% in feed for 21 days) (79). In contrast, in zebrafish larvae, PS-NPs (25 ppm or 25 mg/L for 4 days) increase feeding (80) and in California grunion (*Leuresthes tenuis*) larvae, PE-MPs (15 days exposure) increase feeding rates at low (83 MPs/L), but not high (250 MPs/L) concentrations (81).

MPs disrupt appetite regulation at both peripheral and central levels. In goldfish, reduced food intake induced by exposure to polystyrene MPs or NPs (PS-MPS 0.69 mg/L for 28 days) is associated with decreased levels of orexigenic genes (ghrelin in intestine, hypothalamic neuropeptide Y - NPY - protein content and increased anorexigenic signals (cholecystokinin - CCK, adrenocorticotropic hormone - ACTH, corticotropin-releasing factor - CRF cortisol) (76). Similarly, in pond loach, MP exposure (PE-MPs 1 mg/L for 14 days) down-regulates orexin, a key appetite-stimulating neuropeptide, while upregulating intestinal anorexigenic peptides such as CCK, peptide YY- PYY, and gastric inhibitory polypeptide - GIP, suggesting enhanced satiety signaling and reduced feeding motivation (74). Interestingly, in zebrafish embryos, PS-MP exposure (1.5mg/L, from 6 to 96 hours post-fertilization) increases NPY gene expression, indicating potential life stage-specific responses (82).

MP exposure can also induce behavioral changes relevant to feeding, including reduced swimming activity and reduced predatory performance, leading to reduced foraging and prey consumption, as well as changes in social/shoaling behavior. In fish, sluggish movement, reduced or erratic swimming, and sometimes loss of equilibrium have been shown in several species exposed to MP/NP, including Nile tilapia (MPs 8% in feed for 21 days) (79), walking catfish (MPs 5% in feed for 60 days) (78), crucian carp (*Carassius carassius*) (PS-MPs 0.1 g/L for 24 hours) (51), gilt-head bream (*Sparus aurata*) (PE-MPs, 10% in diet for 21 days) (83), jacopever (10^6^ PS-MPs/L for 14 days) (72), goldfish (1000 MP fibers/L for 45 days) (75), gobies (*Pomatoschistus microps* and *Neogobius melanostomus*) (1000 PE-MPs/L for 37 days; 100 PE-MPs/L for 96 hours) (84, 85), mosquito fish and fathead minnow (*Pimephales promelas*) (25 mg/L PS-MPs for 25 minutes) (86), zebrafish (0.1–20 mg PE-MPs of feed for 28 days; 0.5 - 1.5 mg/L PS-NPs for 7 days) (68, 87) and discus fish (*Symphysodon aequifasciatus*) (PS-NPs 200 µg/L for 96 hours; PE-MPs, 200 µg/L for 30 days) (88, 89). A decrease in performance is accompanied by decreases in levels of neurotransmitters (acetylcholine, dopamine, γ-aminobutyric acid) in the brain of both discus fish (88, 89), and goldfish (0.26-0.69 mg/L PS-MPs or PS-NPs for 28 days) (90), acetylcholine in the intestine and liver of crucian carp (PE-MPs 32 and 64 mg/L for 2 weeks) (91), acetylcholine activity in the liver of common carp (*Cyprinus carpio*) (2.5 PS-MPs/L for 48 or 72 hours) (92), and acetylcholinesterase (AChE) activity in the brain of zebrafish (PVC-MPs, 3-30 µg/L for 20 days) (93). However, in common goby (*P. microps*), MP exposure (0.184 mg/L for 96 hours) does not affect either predatory performance or brain acetylcholine levels (94) and in zebrafish larvae, exposure to PS-MPs (10–100 μg/L from 2 hours to 120 hours post-fertilization) decreases locomotion but increases neurotransmitter (serotonin, dopamine and acetylcholine) levels and activity of AChE (95). Interestingly, some planktivorous species show no clear effects of plastic on behavior, presence on foraging or aggression, further highlighting interspecific variability. For example, in spiny chromis (*Acanthochromis polyacanthus*), MP exposure (PE-MPs 0.025 - 0.1 mg/L for 1–6 weeks) does not affect activity, foraging or aggression (96). Similarly, when exposed to MPs mixed with preys (Daphnia), while common carp, a bottom dwelling omnivore, indiscriminately ingests MPs, leading to reduced predatory performance, catla, a surface feeding planktivore, completely avoids MPs, maintaining high predatory efficiency (2.5 PS-MPs/L for 48 or 72 hours) (92).

It is possible that the effects of MP on feeding - and perhaps other behaviors - might be related to alterations in olfactory and sensory-driven responses. In goldfish, MPs (0.26-0.69 mg/L PS-MPs or PS-NPs for 28 days) impair the olfactory-mediated behavioral responses by causing olfactory bulb injuries, disrupting transmission in olfactory sensory neurons and downregulating genes encoding receptors (90). In zebrafish, exposure to NPs (0.1 mg/L for 45 days) cause structural to the retina, impairing vision (97). The fact that common carp do not discriminate between MPs resembling preys and actual preys, whereas catla do (2.5 PS-MPs/L for 48 or 72 hours) (92) suggests that species-specific sensory mechanisms may influence the effects of MPs, with carp relying more on visual and olfactory cues and catla using more gustatory or tactile cues during prey detection.

Behavioral plasticity and learning may further modulate exposure: for example, bluegill (*Lepomis macrochirus*) (0.02 g MPs in feed per fish for 4–6 days) (98) and golden shiners (*Notemigonus crysoleucas*) (6 mg MP/fish/day via feed for 25 days) (99) reduce plastic ingestion over time when exposed to mixed food and MPs, suggesting learned avoidance. Fish may also mistake MPs for food, actively ingest them, and later expel them, as observed in zebrafish (PS-MPs 0.5mg/L for 3 days; 10,000 PE-MPs/L, for 1, 5, or 10 days) (67, 100).

MP/NP exposure can also alter social behavior, in particular shoaling, a behavior in which fish swim together to reduce anxiety and predation risk. For example, in carp, exposure to NPs induces tighter group cohesion and reduced exploratory behavior (101). Similarly, in jacopever, MP exposure increases shoaling (72), and NP-exposed zebrafish exhibit tighter shoals, which might indicate a stress response (87, 97). In contrast, reduced shoaling behavior is observed in MP-exposed medaka *(Oryzias latipes*) (PS-MPs 0.1 mg/L for 28 days) (102), with MP-exposed (Ps-MPs 0.04 mg/L for 7 days) fish leaving the shoal more frequently than controls (103). In gilt-head bream, control fish are shyer during social interactions and less active during feeding compared to MP-exposed individuals (PE-MPs, 10% in diet for 21 days) (83), which may be associated with increased energetic demands associated with stress responses, inflammation, and impaired nutrient absorption following MP exposure. In another study in gilt-head seabream (10^3^ PE-MPs/L or 4.1 mg/L for 2 hours per day, repeated over 5 consecutive days), fish with high MP ingestion rates exhibit inferior escape responses, which may result from decreased energy efficiency due to ingestion of indigestible particles (104). Together, these studies indicate that MP/NP exposure affects multiple aspects of fish behavior, including shoaling, social interactions, feeding activity, and escape performance, with responses varying among species and exposure conditions.

### 4.2 Amphibians and reptiles

In amphibians, data remain limited and MP ingestion has been documented primarily in larval stages (105). For example, 12 days old axolotl (*Ambystoma mexicanum*) larvae fed zooplankton exposed to Acrylonitrile Butadiene Styrene (ABS) MPs (at a concentration of 200 mg/L for 3 hours) for 5 days consume less prey than controls (106) and feeding rates, survival, and growth decrease in midwife toad (*Alytes obstetricans*) tadpoles exposed to PS-MPs (18, 180, and 1800 MPs/ml for 14 days) (107). Similarly, in African clawed frogs (*Xenopus laevis)*, exposure to 60 mg/L.

MPs for 23 days after hatching leads to particle accumulation in the gut and is associated with reduced feeding rates, altered metabolism, and stress responses (108). Tadpoles of *Xenopus tropicalis* frogs that consume MPs tend to reduce their feeding, likely due to MP accumulation in the digestive tract, which can impair gut function and induce false satiety (109, 110). A field study in China shows that omnivorous tadpoles with labial teeth (*Rana limnochari*) ingest more MPs than filter feeders (*Micohyla ornata* and *M. heymonsi*), and MP accumulation increases with developmental stage (111). However, other studies show no effect of MP on feeding rates of swimming behavior [e.g., *X. laevis* tadpoles (0.125, 1.25, and 12.5 μg/mL PS-MPs from pre-feeding stage to end of the early larval period; 18, 180, 1800 PS-MPs/mL for 229 days, until metamorphosis) (110, 112)]. Italian agile frog (*Rana latastei*) tadpoles are affected by MP exposure, showing reduced growth, activity levels, and high mortality rates even at low MP concentrations, while green toad (*Bufotes balearicus)* tadpoles exhibit little to no effects at low and medium MP concentrations, with only slight impacts at the highest concentration (1, 7 and 50 mg/L for one week after hatching) (113). In addition, Italian agile frog tadpoles actively ingest MPs, while Balearic green toad tadpoles showed less interest in MPs, suggesting interspecies differences in feeding behavior and susceptibility (113).

In reptiles, evidence is sparse and largely restricted to gastrointestinal obstruction rather than mechanistic disruption of digestion, with most data focused on turtles (114). Plastic ingestion has been documented in both marine (115, 116) (117) and freshwater (118) turtles. In both green turtles (*Chelonia mydas*) and juvenile loggerhead turtles (*Caretta caretta*), nutrient uptake decreases with increasing plastic ingestion (116, 119). Green turtles with high gastrointestinal plastic loads show reduced food intake, likely due to satiety or gut blockage (114). High levels of plastic ingestion in turtles are associated with increased mortality risk, as even small amounts can cause gut perforation or impaction leading to death (120). There is also evidence of the presence of plastics in American alligator (*Alligator mississippiensis*) (121), but no functional studies have ever been conducted.

## 5 Digestive function, metabolism, and growth

Ingestion of MPs and NPs disrupts digestive physiology and energy balance in aquatic ectotherms by inducing structural damage to the gastrointestinal tract, altering digestive enzyme activity and gut microbiota, and impairing nutrient digestion and absorption, which in turn disrupts metabolic regulation, energy storage, growth, and overall physiological condition.

### 5.1 Changes in gut structure

#### 5.1.1 Fish

High concentrations of MP/NPs cause intestinal irregularities in fish, including lining/enterocyte damage and increased permeability. A consistent effect across species is intestinal epithelial and enterocyte damage, characterized by breakage and detachment of the epithelium, erosion of villi, enterocyte necrosis and detachment of the lamina propria as seen in several fish, including Nile tilapia (MPs 1 and 10mg/L for 2 weeks; 8% in feed for 21 days; 2000 μg/g in feed for 10 weeks) (79, 122, 123), goldfish (PS-MPs 0.05, 0.5, and 5 mg/L for 28 days; 15 MP per fish for 45 days; 100 and 1000 MP fibers/L for 45 days; 0.5 mg MP/L for 4 days) (75, 124–126), European sea bass (*Dicentrarchus labrax*) (100–500 MP/kg of feed fro 3 weeks; 0.1% in diet for 90 days) (127, 128), sea chub (*Girella laevifrons*) (7–70 mg MP/g fish for 45 days) (129), catla (2.5% PS-MPs in diet for 90 days) (77), wami tilapia juveniles (*Oreochromis urolepis*) (1–100 PE MPs/mL for 65 days) (130), zebrafish (PVC-MPs 3 and 30 µg/L for 20 days; MPs 10 mg and 17.5 mg/L for 5 days; MP 1mg/L for 90 days; PE-MP 0.1, 10, 50, and 500 μg/L for 12 and 24 days; MPs 0.001–10.0 mg for 2 days) (54, 93, 131–133), gilthead seabream (25 mg/kg fish/day in feed for 21 days) (134), common carp (PE-MP 1000 ng/L for 21 days) (135), Japanese medaka (PS-NPs 10, 104, and 106 items/L for 3 months) (136), juvenile orange-spotted groupers (*Epinephelus coioides*) (PS-NPs 300 and 3000 μg/ml for 14 days) (137), rainbow trout (PS-NPs 300 and 3000 μg/ml for 14 days) (138), rohu (*Labeo rohita*) (polylactic MPs 1%, 1.5%, 2% and 2.5% in feed for 90 days) (139), yellow perch (*Perca flavescens*) (8 g HDPE/100 g diet for 9 weeks) (140), grass carp (*Ctenopharyngodon idella*) (100 μg/L PS-NPs for 5 days) (141) [see (142) for more references]. Plastics modulate goblet cell (mucus-secreting intestinal epithelial cells that protect the gut lining, facilitate nutrient passage and maintain barrier integrity) structure and abundance in a species-dependent manner, inducing reduced goblet cell density in largemouth bass (*Micropterus salmoides*) exposed to PS-NPs (10 and 100 μg/L for 7 and 19 days) (143), and induce goblet cell hypertrophy in NP-exposed guppy (*Poecilia reticulata*) (100 and 1000 μg/L for 28 days) (144). Similarly, MP/NPs increase the secretory activity of mucus cells in intestine (and buccal cavity, skin and gills) in goldfish (100 and 1000 MP fibers/L for 45 days) (75) and marine medaka (*Oryzias melastigma*) (2.5 µg/mL PS-MPs for 14 days) (145). However, a few studies report no effects of MP on gut structure [e.g., pacu (*Piaractus mesopotamicus*) (PE-MPs 0.1% in feed for 15 days) (146), gilt-head seabream (0.1 g /kg of fish/day for 45 days) (147), turbot (*Scophthalmus maximus*) (NPs, 2% of feed for 3 and 9 weeks) (148)].

MP exposure induces intestinal inflammation, as evidenced by activation of inflammatory pathways/cytokines. MP exposure increases the secretion of tumor necrosis factor-alpha (TNF-α), interferon-gamma (IFN-γ), and interleukin-6 (IL-6) in guppy (144), common carp (PE-MP 1000 ng/L for 21 days) (135) and TNF-α and IFN-γ in marine medaka (MPs 1 and 10mg/L for 2 weeks) (122), and intestinal gene expression of TNF-α and interleukin-1 (IL-1β) in gilthead seabream (PS-MPs; 1–20 μm; 25, or 250 mg/kg fish/day for 21 days) (149) and IL-1β in koi carp (PS-NPs 0.02, 0.2 and 2 mg/L for 7 days) (150). In seabream, MP exposure also decreases the expression of anti-inflammatory cytokines (i.e., interleukin-10 (IL-10) (25 mg/kg fish/day in feed for 21 days) (134), whereas in zebrafish, MPs increase both mRNA and protein levels of IL-1α, IL-1β, and IFN in the gut (MPs 1000 μg/L for 14 days) (151).

In addition to cytokine responses, MP/NP exposure affects innate immune defenses by reducing the activity of enzymes such as lysozyme, an antimicrobial enzyme that breaks down bacterial cell walls. For example, decreased lysozyme activity is seen in orange-spotted grouper (PS-NPs, 300 and 3000 μg/ml for 14 days) (137) and yellow croaker (*Larimichthys crocea*) (100 nm NPs, 10, 10^4^ and 10^6^ mg/L for 14 days) (152), suggesting impaired innate immune function in the gut.

MP/NPs can also impair liver structure and function. For example, in turbot, long-term exposure to either MPs or NPs causes hepatocyte damage and increases the expression of inflammatory cytokine expression, with NPs producing stronger effects than MPs (diet containing 2% MPs or NPs for 3 and 9 weeks) (148). Similar hepatic damage and inflammatory responses have been reported in other fish [e.g., zebrafish (PE-MP 0.1, 10, 50, and 500 μg/L for 12 and 24 days) (132), Nile tilapia (PS-NPs 100 μg/L for 14 and 21 days) (153), carp (PS-MPs 1000 ng/L for 21 days) (154), rainbow trout (0.5, 2 and 5% in feed for 6 weeks) (73), orange-spotted grouper (PS-NPs 300 and 3000 μg/ml for 14 days) (137)] following MP exposure.

#### 5.1.2 Amphibians and reptiles

Histopathological and morphological effects of MPs have also been reported in several amphibians (105). Post metamorphic semi-aquatic black-spotted pond frogs (*Pelophylax nigromaculatus*) exposed to 18, 180 and 1800 PS-MPs/ml (10 μm) for 6 weeks (frogs were kept in glass cage with a split aquatic and terrestrial section, and MPs were present in the aquatic section) display show altered gastrointestinal morphology, with longer stomachs and shorter intestines (155), while aquatic *X. laevis* larvae exposed to 60 mg/L of PE-MPs (particle size: 34–50 μm) for 23 days exhibit increases in gut length and mass (108). The contrasting responses may reflect differences in species, developmental stage, habitat and exposure route, as well as variation in polymer type, particle size, and exposure duration.

In *Rhinella arenarum* tadpoles, MP (PE-MPs 60 mg/L for 30 days) ingestion causes mechanical damage abrasion of the intestinal walls (156). Similarly, bullfrog tadpoles (*Lithobates catesbeiana*) exposed to 100 and 1000 μg/L of PS-MPs for 28 days display structural damage to intestinal tissues, including the separation of the intestinal muscle layer from epithelial cells, and enterocyte deformation and vacuolization (157). Histopathological changes are also observed in the liver of *Physalaemus cuvieri* tadpoles exposed to 60 mg/L PE-MPs for 7 days (158).

Evidence in reptiles is very limited. Marine plastic debris have been found in the gastrointestinal tract of marine turtles. In several marine turtles, large amounts of plastic (mostly large debris) accumulate in the digestive system and can cause physical blockage, obstruction, or puncture, impairing the movement of intestinal contents (159, 160). In green turtles, ingested plastic has been associated with perforation of the gastrointestinal tract, leading to bacterial infections, ulcers, and necrosis (161).

### 5.2 Nutrient digestion and absorption

#### 5.2.1 Fish

A consistent effect of MP/NP exposure is the disruption of digestive enzymes, particularly proteolytic (trypsin, chymotrypsin), lipolytic (lipase), and carbohydrate-digesting (amylase) enzymes. Decreases in these enzymes have been reported in multiple species, including orange-spotted grouper, in which NPs reduced lipase, trypsin, and lysozyme activities (PS-NPs 300 and 3000 μg/ml for 14 days) (137), in guppy, where MPs lowered trypsin, chymotrypsin, amylase, and lipase activities (144), and yellow croaker, for which NP exposure reduce lipase and trypsin activities (152). Similar reductions in several digestive enzymes following MP exposure (0.19 mg MPs/day via feed for 42–46 weeks) have also been observed in redtail notho (*Nothobranchius guentheri*) (162), in golden pompano (*Trachinotus blochii*) (10 μg/L, 100 μg/L and 1000 μg/L PE-MPs for 14 days) (163), and brown trout (*Salmo trutta*) (164). In contrast, some species exhibit increased enzymatic activity, as observed in goldfish (intestinal lipase, amylase, and protease) (1, 10, and 100 mg PS-MPs/kg of diet for 21 days) (165) and in peled (*Coregonus peled)* larvae (trypsin, α-amylase, and bile salt-activated lipase) (PS-MPs 5, 50 and 500µg/L for 24 hours and 6 days) (166).

In addition to digestive enzyme disruption, MPs reduce nutrient absorption by impairing intestinal transport processes. For example, in pond loach, MP exposure (PE-MPs 1 mg/L for 14 days) downregulates the expression of some intestine glucose transporters, which may impair glucose absorption and overall nutrient metabolism (74). Similarly, in rohu (139) (polylactic MPs 1%, 1.5%, 2% and 2.5% in feed for 90 days) and catla (167) (polylactic MPs 0.5 %, 1 %, 1.5 %, 2 %, and 2.5 % in feed for 90 days), MP exposure reduces protein and mineral digestibility, resulting in reduced assimilation efficiency and malnutrition.

#### 5.2.2 Amphibians and reptiles

There is no direct evidence for digestive enzyme disruption in either amphibians or reptiles. Given the consistent structural damage observed in amphibian larvae, impairment of enzymatic and absorptive function is likely. In reptiles, no studies have directly examined the effects of plastics on gastrointestinal enzymes, but severe gastrointestinal blockage has been reported in turtles, suggesting disruption of digestive processes.

### 5.3 Metabolic regulation and nutrient storage

Once nutrients are absorbed, MPs and NPs disrupt systemic metabolic regulation across hepatic, endocrine, and oxidative stress pathways.

#### 5.3.1 Fish

In gilthead seabream, polystyrene microplastics (PS-MPs; 1–20 μm; 25, or 250 mg/kg fish/day for 21 days) upregulate hepatic genes involved in lipid synthesis and storage (e.g., peroxisome proliferator-activated receptor alpha - PPARγ, fatty acid synthase - Fasn), without affecting genes associated with lipid catabolism (e.g., hepatic lipase - HL) or transport (e.g., fatty acid binding protein - Fabp1), suggesting a shift toward lipid accumulation (149). In juvenile European seabass, exposure to environmentally weathered MPs (0.5 mg/kg and 1 mg/kg of feed for 5 days) leads to depletion of lipid metabolites (e.g., glycerophospholipids, diacylglycerols, phosphatidylserines, and unsaturated fatty acids) in intestinal tissues (168). In larval zebrafish, exposure to NP leads to decreased body glucose levels and a reduction in the size of the insulin-producing pancreatic islets, associated with upregulated genes related to gluconeogenesis (20 mg/L PS-NPs for 48 hours) (169). However, in crucian carp, exposure to high concentrations of MPs increases blood glucose levels (PE-MPs 32 and 64 mg/L for 2 weeks) (170).

Alterations in liver-associated enzymes, together with changes in oxidative stress indicators, indicate that plastics can disrupt liver function and metabolic balance. In Putitor mahseer (*Tor putitora*), MPs (PE-MPs 0.1, 1, and 10 mg/L for 15 and 30 days) increase plasma levels of aspartate aminotransferase, alanine aminotransferase, alkaline phosphatase, and lactate dehydrogenase, consistent with hepatic stress or damage (171). Similar increases in these metabolic enzymes have been reported in crucian carp after MP exposure (PE-MPs 32 and 64 mg/L for 2 weeks) (170) and in goldfish after NP exposure (1, 10, and 100 mg PS-MPs/kg of diet for 21 days) (165). In pearl spot (*Etroplus suratensis*), NP exposure (0.2, 2, and 4 mg/L PS-NPs for 14 days) increases hepatic levels of ALT (alanine aminotransferase), serum glutamic-oxaloacetic transaminase (SGOT), and serum glutamic pyruvic transaminase (SGPT) - enzymes involved in the metabolism of amino acids derived from proteins - suggesting disruption of hepatic homeostasis and that a potential greater use of amino acids for energy demands under stress (172). In contrast, in redtail notho, MP exposure (0.19 mg MPs/day via feed for 42–46 weeks) reduces hepatic activities of amylase, aminotransferase, alkaline phosphatase and glycogen synthase (GS) activities (162), indicating suppressed digestive capacity.

MP exposure also frequently disrupts antioxidant defense systems and promotes oxidative stress, resulting in the accumulation of reactive oxygen species (ROS) and subsequent cellular damage. In gilthead seabream, MPs (PS-MPs; 1–20 μm; 25, or 250 mg/kg fish/day for 21 days) alter both enzymatic (increased catalase and superoxide dismutase (SOD) mRNA levels, enzymes that convert ROS into less harmful molecules) and non-enzymatic (glutathione, an intracellular antioxidant that neutralizes harmful molecules and supports antioxidant enzyme activity) antioxidant components in the intestine, leading to elevated ROS levels, indicative of oxidative damage (149). Similar increases in intestine and liver SOD and catalase activities have been reported in other fish, including zebrafish (PE-MP 0.1, 10, 50, and 500 μg/L for 12 and 24 days; PS-MPs 200 and 2000 μg/L for 7 days) (132, 173), crucian carp (PE-MPs 32 and 64 mg/L for 2 weeks) (170) and Asian catfish (*Heteropneustes fossilis*) (100, 500 and 1000 μg/L PE-MPs for 7 days) (174). In contrast, NP exposure can suppress antioxidant defenses in some species. For example, in stone moroko (*Pseudorasbora parva*), NP exposure (10 mg/L for 7 days) decreases SOD and catalase activities, likely because excessive ROS production overwhelms antioxidant capacity and direct NP - interactions impair enzymatic function (175). Similarly, in pearl spot, NP exposure (0.2, 2, and 4 mg/L PS-NPs for 14 days) reduces antioxidant levels, including SOD and catalase (172).

Disruption of metabolic regulation leads to altered allocation of energy reserves in liver and muscle tissues. In yellow perch, dietary exposure to MPs (8 g HDPE/100 g diet for 9 weeks) alter nutrient metabolism pathways, resulting in decreased protein and ash content, increased hepatosomatic index, hepatocyte size, and liver glycogen levels, and reduced hepatic lipid content (140). Decreased protein and ash content with increased moisture and fat are also observed at high MP exposure levels (2.5% PS-MPs in diet for 90 days) in catla (77). MP/NPs reduce carbohydrate, protein, and lipid levels in rohu (1, 3, and 5 mg/L PE-MPs for 60 days) (176), and glucose and triglyceride contents in channel catfish (*Ictalurus punctatus*) larvae (5, 10, 25 and 50 mg/L PS-NPs for 24 h or 48 h) (177). In zebrafish embryos, exposure to PE pellets (10 and 30 g/L for 24 or 48 hours) upregulates the expression of genes related to lipid metabolism and adipogenesis (178). In contrast, in goldfish, high MP concentrations (40 mg/L for 28 days) increase triglyceride and cholesterol levels (179). In pearl spot, NP exposure (0.2, 2, and 4 mg/L PS-NPs for 14 days) increases glucose, total cholesterol, SGOT, SGPT, and ALP levels and decreases total protein (172). In grass carp, exposure to PS- MPs (100 μg/L and 400 μg/L for 30 days) downregulates genes related to metabolism and insulin signaling and activates pathways associated with insulin resistance and type 2 diabetes, indicating disruption of glucose metabolism (180). In golden pompano, PS-MP exposure (100.0 μg/L, and 1000.0 μg/L for 14 days) inhibits genes involved in lipid metabolism (including linoleic acid metabolism, fatty acid elongation, and fatty acid degradation) and carbohydrate metabolism (starch and sucrose pathways) (163). In zebrafish, MPs (200 µg/L and 1 mg/L for 30 days) disrupt lipid and fatty acid metabolism (such as downregulation of triglyceride, cholesterol esters and phospholipid synthesis pathways) (181).

#### 5.3.2 Amphibians and reptiles

Data on the effects of plastics on metabolic regulation and nutrient storage in amphibians and reptiles is very limited. In *Rhinella arenarum* tadpoles, exposure to MPs (PE-MPs 60 mg/L for 30 days) leads to hepatocyte vacuolization, an indication of cellular stress and hepatotoxicity (156), suggesting impaired liver function and potentially disrupted metabolic processes. In Zhenhai brown frog (*Rana zhenhaiensis*), exposure to MPs (50 mg/L polypropylene, polystyrene or polyethylene MPs form start of swimming and feeding to when forelimbs emerged) leads to enlarged intercellular spaces in the liver, indicative of tissue damage, downregulates antioxidant genes (e.g., glutathione peroxidase 1- GPX1- and glutathione S-transferases -GST), thereby promoting the overproduction of reactive oxygen species (ROS), and disrupts genes involved in energy metabolism, including glycolysis and the tricarboxylic acid cycle (182). In East Asian bullfrog (*Hoplobatrachus chinensis*), MP exposure (PE-MPs 10 mg/L for 4 days) initially induces an adaptive increase in antioxidant enzymes such as superoxide dismutase (SOD) and catalase, but prolonged exposure ultimately exhausts these defenses, resulting in decreased enzyme activity (183).

### 5.4 Growth and physiological condition

The combined disruption of nutrient acquisition, metabolic regulation, and energy storage ultimately impairs growth and physiological condition.

#### 5.4.1 Fish

Growth inhibition in fish is commonly observed following MP/NP exposure and is often dose-dependent, occurring particularly at moderate-to-high MP concentrations and longer exposure durations. Dose-dependent reductions in weight gain and growth rate have been reported in several fish species, including orange-spotted grouper (PS-NPs 300 and 3000 μg/ml for 14 days) (137), catla (2.5% PS-MPs in diet for 90 days) (77), wami tilapia (1–100 PE MPs/mL for 65 days) (130), Nile tilapia (10 mg/L PA-MPs for 42 days) (184), golden pompano (10, 100 and 1000 μg/L PS-MPs for 14 days) (185), fathead minnows (4 -16 500 μm PE-MPs per fish per day for 28 days) (186), grass carp (100 μg/L PS-NPs for 5 days) (141), walking catfish (MPs 5% in feed for 60 days) (78) and zebrafish (MPs 1mg/L for 90 days; PS-MPs 10-100 μg/L for 5 days) (54, 187).

In many fish, MP and NP exposure disrupts the growth hormone (GH)/insulin-like growth factor (IGF) axis, a key endocrine pathway that regulates somatic growth, nutrient utilization, metabolism, and tissue development. In this axis, GH secreted by the pituitary stimulates hepatic and peripheral production of IGF, which mediates many of the growth-promoting effects of GH through binding to IGF receptors in target tissues (188). MP exposure decreases expression of genes of critical components of this axis, including IGF-I, IGF-1 receptor (IGF-IR), IGF binding proteins and growth hormone receptor (GHR) [e.g., marine medaka (20 μg/L PS-MP for 150 days) (189), Nile tilapia (10 mg/L PA-MPs for 42 days) (184), goldfish (0.5 mg/L PVC-MPs for 4 days) (126), zebrafish larvae (PS-MPs 10-100 μg/L for 5 days; MPs 500 μg/L 0–30 days post fertilization) (187, 190) and pearl spot (PS NPs 2-4mg/L for 14 days) (172)]. These findings suggest that impairment of the GH/IGF axis by MPs and NPs inhibits growth and may alter energy metabolism in fish.

In zebrafish larvae, MP exposure (PS-MPs 10-100 μg/L for 5 days) disrupts the hypothalamic-pituitary-thyroid (HPT) axis, a key neuroendocrine system that regulates thyroid hormone production and controls growth and metabolism in fish (187). Under normal conditions, the hypothalamus releases thyrotropin-releasing hormone (TRH), which stimulates the pituitary to secrete thyroid-stimulating hormone (TSH). TSH then acts on thyroid cells to stimulate the synthesis and release of thyroid hormones, which exert negative feedback on both the hypothalamus and pituitary (191). MP exposure in zebrafish larvae increases thyroid hormone levels and reduces expression of TSH and thyroid hormone receptors (187), suggesting a disruption of thyroid hormone signaling, which may contribute to the metabolic and growth impairments observed following MP exposure.

However, some studies report no significant effects of long-term exposure to low MP/NP concentrations, including medaka (1.5, and 3 PE fibers/fish/day for 21 days; 50 and 500 μg/L PS-MPs for 150 day) (192, 193), juvenile barramundi (*Lates calcarifer*) (PE fibers 1% in diet for 56 days) (194), three-spined stickleback (*Gasterosteus aculeatus*) (MP fibers 1000, and 10,000 fibers per gram of food for 30 days) (195) and juvenile turbot (diet containing 2% MPs or NPs for 3 and 9 weeks) (148).

It is noteworthy that in some species, compensatory growth may occur. Compensatory growth refers to accelerated growth in previously fasted fish, relative to continuously fed individuals, that occurs after refeeding (196). For example, in juvenile spiny chromis, an initial week of acute exposure where food is replaced by MPs (PE-MPs 0.025 - 0.1 mg/L for 1–6 weeks) induces significant weight loss. However, during the subsequent six-week of chronic exposure - where fish are fed a normal food ration alongside MPs - the fish that had lost mass perform compensatory growth, “catching up’” with the rest of their cohort. By the end of this period, there is no significant difference in body mass between the treatment groups and the control (96).

#### 5.4.2 Amphibians and reptiles

The effects of MPs on amphibian growth and development are variable and species-specific. A broad meta-analysis across aquatic organisms shows that bisphenol A (BPA, a plastics-derived endocrine disruptor) negatively affects development and growth, with amphibians being particularly sensitive compared to other vertebrate groups (197). Some studies report reduced larval growth and developmental rates following MP exposure [e.g., *X. laevis* (60 mg/L PE-MPs for 7 weeks, from hatching to metamorphosis) (105); Italian agile frog (1, 7 and 50 mg/L for one week after hatching) (113); Zhenhai brown frog (50 mg/L polypropylene, polystyrene or polyethylene MPs form start of swimming and feeding to when forelimbs emerged) (182)]; Asian bullfrog (*Hoplobatrachus chinensis*) (PE-MPs 10 mg/L for 4 days, mid-larval development, prior to limb formation and metamorphosis) (183)]. In contrast, some studies show no significant effects of MPs on growth [e.g., *X. laevis* tadpoles (0.125, 1.25, and 12.5 μg/mL PS-MPs from pre-feeding stage to end of the early larval period) (112); green toad tadpoles (1, 7 and 50 mg/L for one week after hatching) (113)] and some studies report increased growth following MP exposure, such as in Italian agile frog tadpoles (1, 7 and 50 mg/L for 4 weeks, mid-larval development prior to metamorphosis) (198) and wood frog (*Rana sylvatica*) larvae (mixture of polystyrene and polyethylene terephthalate at 0.069 g/L and 0.691 g/L during the entire aquatic phase from embryo to metamorph) (199).

Although these findings appear contradictory, direct comparisons among studies are complicated by major differences in experimental design. For example, reduced growth in Italian agile frog tadpoles was observed following a one-week exposure immediately after hatching (113) whereas increased growth was reported after a 4-week exposure during mid-larval development (198), suggesting that developmental stage and exposure duration may influence the response to MPs. Similarly, growth inhibition in *Xenopus laevis* was reported following continuous exposure from hatching through metamorphosis (105), whereas no effects were observed when exposure was limited to the early larval period (112). These observations suggest that the effects of MP/NPs on amphibian growth may depend on the timing and duration of exposure relative to key developmental transitions.

Evidence in reptiles remains very limited but suggests potential negative effects of plastic ingestion on body condition. High gastrointestinal plastic loads have been associated with underweight conditions and emaciation, likely due to reduced nutrient assimilation and gut obstruction (114).

### 5.5 Microbiota

#### 5.5.1 Fish

Gut microbiota alterations have been reported following MP/NP exposure in several fish species. Across species, plastics consistently reduce microbial diversity and alter community structure. These changes have been documented in several species, including rainbow trout (PS-NPs 300 and 3000 μg/ml for 14 days; PS-MPs 5000 μg/ml for 14 days) (138, 200), marine medaka (2.5 μg/L and 2.5 mg/L PS-MPs for 1 month; 2.5 µg/mL PS-MPs for 14 days) (145, 201), medaka (PS-MPs 0.1 mg/L for 28 days) (102), common carp (0.2 or 2 mg/L PVC-MPs for 3 weeks; PS-MPs 1000 ng/L for 21 days; PS-NPs 0.02, 0.2 and 2 mg/L for 7 days) (150, 202, 203) grass carp (*Ctenopharyngodon idellus*) (100 μg/L PS-NPs for 5 days) (141), zebrafish (PS-MPs 50 and 100mg/L for 15 days; PE-MPs 50 mg/L for 14 days; MPs 10 mg and 17.5 mg/L for 5 days; 200 µg/L and 1 mg/L for 30 days; 10,000 PE-MPs/L, for 1, 5, or 10 days) (100, 131, 181, 204, 205), discus (PS-NPs 200 µg/L for 96 hours) (88), guppy (100 and 1000 μg/L for 28 days) (144), orange-spotted groupers PS-NPs 300 and 3000 μg/ml for 14 days) (137), largemouth bass (NPs 10 and 100 μg/L for 7 and 19 days) (143), European sea bass (0.5 mg/kg and 1 mg/kg of feed for 5 days; polypropylene MPs 10% in feed for 60 days) (168, 206), fathead minnows (4–16 PE-MPs per fish per day for 28 days) (186), barramundi (PE fibers 1% in diet for 56 days) (194), turbot (2% MPs or NPs in feed for 3 and 9 weeks) (148) and golden pompano (100.0 μg/L, and 1000.0 μg/L for 14 days) (163).

In many cases, MP/NPs increase the abundance of opportunistic pathogens such as *Vibrio* spp. For example, in orange-spotted grouper, NPs increase pathogenic taxa such as *Vibrio* and *Aliivibrio* (137). Similarly, in grass carp, MP exposure increases Proteobacteria (opportunistic pathogens) and decrease in beneficial bacteria such as *Bacillus* (141).

Microbial disruption is linked to impairment of gut–organ communication axes, including the gut–brain, gut–liver, and gut–gill axes, suggesting systemic consequences beyond the intestine. For example, in marine medaka (PS-MPs 0.2 mg/L for 21 days), disruption of the gut–gill axis - via impaired intestinal integrity, microbial balance, and gill function - compromises nutrient absorption, ion regulation, and respiration, ultimately leading to impaired metabolic function (207). In zebrafish, exposure to polyglycolic acid (PGA) - which breaks down into biodegradable MPs - (1 or 100 mg/L for 28 days) induces gut dysbiosis characterized by an overrepresentation of Gram-negative bacteria, a source of lipopolysaccharide (LPS). Disruption of intestinal barrier integrity facilitates LPS translocation into the circulation, triggering systemic inflammation and hepatic dysfunctions that propagate along the gut–liver–brain axis, leading to neuroinflammation associated with reduced 5-hydroxytryptamine (5-HT) levels, anxiety-like behavior, and cognitive impairment (208).

#### 5.5.2 Amphibians and reptiles

In amphibians, MP exposure can alter gut microbial communities, although the effects differs among species. Tadpoles of Asiatic toad (*Bufo gargarizans*) (0.55 ng/L-2.5 μg/L for 1 μm MPs and 0.55 μg/L - 2.5 mg/L for 10 μm MPs for 3 weeks, before the onset of metamorphic climax) (209) and bullfrog (100 and 1000 μg/L of PS-MPs for 28 days, early to mid-larval development, before the onset of metamorphosis) (157) show reduced microbial diversity and shifts in community composition after exposure. In Asian bullfrog (PE-MPs 10 mg/L for 4 days, mid-larval development, prior to limb formation and metamorphosis) (183) and Zhenhai brown frog (50 mg/L MPs form start of swimming and feeding to when forelimbs emerged) (182), MPs promote potentially harmful bacteria, while reducing beneficial bacteria involved in immunity and nutrient absorption (183). In black-spotted pond frogs exposed to 18, 180 and 1800 PS-MPs/ml (10 μm) for 6 weeks, microbiota changes are related to differential sensitivity among bacterial taxa, suggesting selective effects on microbial populations (155). In contrast, leopard frog (*Lithobates pipiens*) tadpoles show little to no detectable disruption of gut microbiota after exposure (polyester MPs fibers 10 μg/L and 40 μg/L for 32 days in early larval stages) (210).

Data in reptiles remain very limited. In hatchlings of the Chinese soft-shelled turtle (*Pelodiscus sinensis*), PS-NPs exposure (1 mg/L from egg development to 6 month after hatching) reduces microbial diversity and increases pathogenic bacteria while reducing beneficial bacteria, indicating dysbiosis (211).

## 6 Interactions of plastics with environmental factors

The effects of plastics on feeding, growth, metabolism, and tissue integrity are modulated by environmental conditions. Among abiotic factors, temperature and salinity are the most frequently studied. To our knowledge, other environmental variables such as oxygen availability, pH, turbulence and turbidity, have not been examined. Interactions between plastics and chemical pollutants (e.g., metals, organic contaminants) are not considered here, as this represents a distinct and extensive body of literature beyond the scope of this review [see reviews such as (16, 212, 213)].

### 6.1 Fish

#### 6.1.1 Temperature

Temperature is the most extensively studied environmental modulator of plastic effects on feeding-related processes in fish. Elevated temperature can modulate physiological and behavioral responses in fish, although responses vary across species and biological processes considered.

In round goby (*N. melanostomus*), combined exposure to high temperatures (26 °C *vs* 18 °C) and MPs (1000 PE-MPs/L for 37 days) produces stronger reductions in feeding performance than either stressor alone (84). Similarly, in common goby, high temperatures (25 °C *vs* 20 °C) reduce hunting efficiency and increase mortality, and these effects are exacerbated by the presence of MPs (0.184 mg/L for 96 hours) (94). In zebrafish, combined exposure to NPs (PS-NPs 1mg/L for 4 days) and high temperatures (30 °C compared to 28 °C) increases the severity of hepatic (214) and brain (215) damage as well as disruption of circadian rhythms - which regulate locomotor activity, foraging behavior, and metabolic processes (215). In Nile tilapia, MP exposure (polyamide MPs 10 mg/L for 15 days) at high temperatures (33 or 36 °C compared to 30 °C) increases ingestion of MPs and intensifies gill and intestinal histopathological damage ( (216).

In larvae of pejerrey (*Odontesthes argentinensis*) exposed to MPs (PS-MPs 40 and 400 µg/L for 96 hours) at 22 and 26 °C, high temperature exacerbates MP-induced oxidative stress (217). Similarly, in Nile tilapia subjected to three temperatures (30, 32, and 34 °C) with or without polyvinyl chloride (PVC) NPs (10 mg/L for 4 days), co-exposure to high temperatures and NPs induces a synergistic interaction, as when combined, these two stressors produce effects that exceeded the sum of their individual effects (218). These included increases in the levels of plasma total proteins, aspartate aminotransferase (AST), alanine aminotransferase (ALT), alkaline phosphatase (ALP), and liver and brain activity levels of oxidative stress biomarkers such as catalase (218).

In discus fish exposed to MPs (200 μg/L for 30 days) at 28 °C and 31 °C, individuals at the higher temperature exhibit greater accumulation of MPs in their bodies, suggesting an increased food intake (89). Interestingly, MP exposure reduces predatory performance at 28 °C but not 31 °C, suggesting that high temperatures - may partially counteract the negative effects of MPs prey capture (89).

Overall, these findings show that high temperatures can modulate plastic toxicity in fish, often enhancing effects on feeding, metabolism, and tissue damage, although interactions may be synergistic or antagonistic depending on context.

#### 6.1.2 Salinity

Compared with temperature, fewer studies have examined salinity–plastic interactions. However, available evidence indicates that salinity could influence the fate and effects of plastics in fish gut.

In Mozambique tilapia (*Oreochromis mossambicus*), fish exposed to higher salinities accumulate more MPs (200 μg/L polyamide MPs for 15 days) in their gastrointestinal tracts and display more severe damage to intestinal tissue (epithelium breakage and enterocyte vacuolization) compared to those in freshwater (219). High salinity also increases the toxicity of copper oxide NPs (CuO NPs 5mg/L for 6 days), due to higher accumulation in the liver and more severe alterations in detoxification pathways than exposure to NPs in freshwater (220). These more severe effects are likely due to increased drinking high-salinity environments (as marine fish continuously drink seawater to compensate for water loss), leading to a higher intestinal intake of NPs, which are then absorbed into the bloodstream and deposited in the liver (220).

A comparable salinity-dependent pattern is observed in Javanese medaka (*Oryzias javanicus*) larvae - a euryhaline fish that adapts to both freshwater and in seawater -, where gut retention time of MPs (PS-MPs 0.25 mg/L for 24 hours) is shorter in seawater than in freshwater, a difference likely linked to salinity-driven increases in water intake, resulting in higher gastrointestinal fluid turnover and faster particle transit. However, despite reduced retention time in seawater, fish accumulate a greater number of MP, likely due to increased water intake (221).

### 6.2 Amphibians and reptiles

There is no direct evidence linking microplastic (MP) exposure and environmental factors with regard to feeding, growth, or metabolic performance in amphibians. However, available studies indicate that environmental factors, particularly temperature, can modulate other physiological functions to MPs.

In Japanese tree frog (*Dryophytes japonicus*) tadpoles, high temperatures (30 °C) reduce mortality and lower the incidence of hindlimb deformities caused by MPs compared to lower temperature (22 °C). At high (but not low) temperatures, MP-exposed tadpoles exposed to 180 MP/mL polystyrene MPs (from free-swimming larvae until the completion of metamorphosis) develop longer intestines, potentially as a compensatory response to reduced energy absorption (222). In contrast, in bullfrog tadpoles, higher temperatures (32 °C *vs*. 25 °C) intensify the effects of polystyrene microplastics (PS-MPs) (100 and 1000 μg/L for 28 days, early to mid-larval development, before the onset of metamorphosis) in the intestine, increasing antioxidant responses (e.g., increased levels of glutathione), immune enzyme activities (e.g., increased lysozyme levels), intestinal damage, and increased levels of pro-inflammatory gene expression (e.g., TNF-α, IL-1β, and IL-8). These combine stressors also alter gut microbiota, reducing microbial diversity, indicating intestinal dysbiosis (157).

To our knowledge, no studies have yet examined interactions between MP exposure and environmental factors in reptiles.

## 7 Conclusion and future perspectives

**Figure 1:**
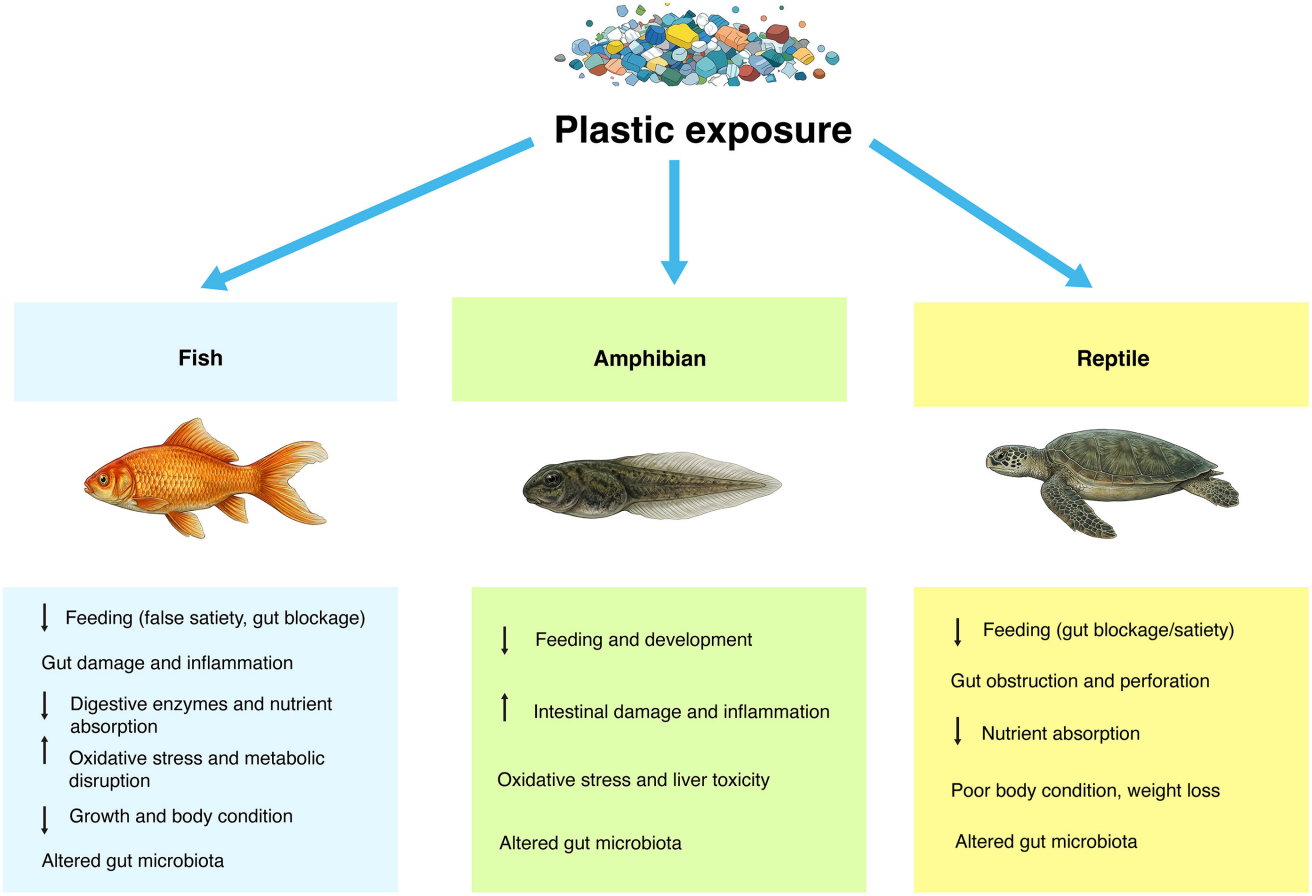
Major known effects of plastics on feeding, digestive physiology and nutrient metabolism in aquatic ectothermic vertebrates (image was created using Adobe Firefly and Adobe illustrator).

Plastic pollution is a widespread stressor in aquatic ecosystems. As summarized in **Table 1** and **Figure 1**, plastics affect multiple components of energy balance in aquatic ectothermic vertebrates, including feeding, digestion, metabolism, and growth. Evidence is strongest for fish, whereas amphibians remain less studied and data for reptiles are largely limited to observational reports.

**Table 1:** Summary of major effects of plastic in aquatic ectothermic vertebrates.

Biological Process	Fish	Amphibians	Reptiles
Exposure and Uptake	High ingestion via water, sediment, and food (preys) Translocation to liver, brain, and gonads in particular small NPs)	Mostly ingestion in larval stages Accumulation in the gut	Some evidence of ingestion (mainly macro- and microplastics) in turtles Limited mechanistic data
Food Intake and Feeding Behavior	Mostly reduced feeding (false satiety, gut blockage) Altered appetite regulation (↓ orexigenic, ↑ anorexigenic signals) Impaired predation and foraging Some increases at low doses or early life stages	Variable Often reduced feeding due to gut accumulation and false satiety Species- and stage-dependent	Reduced feeding linked to gut obstruction and satiety Largely inferred from field observations
Behavioral Effects	Reduced swimming, altered shoaling, impaired predator-prey interactions Neurotransmitter disruption Altered sensory (olfactory) function	Reduced activity and feeding-related behaviors Limited data	Largely unknown
Gut Structure and Integrity	Epithelial damage, villi erosion, inflammation, altered mucus production and increased permeability	Intestinal damage, altered gut morphology (length, structure), tissue abrasion	Physical blockage, perforation, and lesions (mainly from larger plastics)
Digestive Function	Altered digestive enzymes (↓ trypsin, lipase, amylase) Impaired nutrient transport and absorption	Likely impaired digestion (limited direct evidence); Inferred from structural damage	No direct data; Obstruction likely impairs digestion
Microbiota	Reduced diversity, dysbiosis, ↑ and opportunistic pathogens; Disruption of gut-organ axes (gut-liver-brain)	Altered microbiota composition Reduced diversity Increased pathogenic bacteria	Very limited data Some evidence of dysbiosis in turtles
Metabolism and Physiology	Disrupted hepatic function, oxidative stress, and altered lipid/glucose metabolism Endocrine disruption (thyroid, insulin pathways) Energy reallocation	Oxidative stress, hepatotoxicity, disrupted energy metabolism pathways (limited studies)	Very limited mechanistic data Likely impaired condition due to reduced nutrient assimilation
Growth and Condition	Often reduced growth and condition Altered GH/IGF axis Sometimes compensatory growth or no effect at low doses	Variable: reduced, unchanged, or increased growth depending on species and conditions	Reduced body condition and increased mortality risk linked to plastic ingestion
Environmental Interactions	Strong modulation by temperature and salinity (synergistic or antagonistic effects)	Limited but emerging evidence (temperature modifies MP effects)	No data on interactions with environmental factors

Across taxa, plastic exposure often alters feeding processes, most often resulting in reduced food intake. This reduction is primarily linked to false satiety, gastrointestinal obstruction, and behavioral disruption. In fish, decreased feeding is associated not only with physical factors such as gut filling, but also with changes in neuroendocrine appetite regulation and sensory function, indicating centrally mediated responses. Amphibians show similar reductions in food intake, although current evidence is largely descriptive and does not address behavioral, sensory, or endocrine mechanisms. In reptiles, reduced feeding has mainly been linked with physical blockage of the gastrointestinal tract.

Plastics also impair digestive function through structural and functional changes in the gastrointestinal system. In fish, exposure is associated with epithelial damage, inflammation, altered mucus production, and disruption of digestive enzyme activity and nutrient transport, indicating impaired gastrointestinal function that likely reduces nutrient absorption efficiency. Amphibians exhibit comparable histological alterations, suggesting reduced digestive capacity, although direct links to digestive function remain limited. In reptiles, available data are largely restricted to physical damage and obstruction, with little information on digestive physiology or nutrient processing.

Disruption of the gut microbiota also occurs across groups. In fish, plastic exposure often reduces microbial diversity, alters community composition, and increases opportunistic pathogens while reducing beneficial taxa. In amphibians, responses are more variable: some studies report reduced diversity and increases in potentially harmful bacteria during larval stages, whereas others show little or no effect, suggesting species- and stage-specific responses. In reptiles, data are scarce, but current evidence indicates reduced diversity and increased pathogenic bacteria, consistent with dysbiosis, although its consequences remain unclear.

At the systemic level, fish show clear alterations in metabolic regulation, including changes in hepatic function, oxidative stress pathways, and endocrine signaling (e.g., thyroid and glucose-related pathways), leading to shifts in nutrient metabolism and energy allocation. Amphibians show evidence of oxidative stress and metabolic disruption without well-defined mechanistic pathways. Comparable data are largely lacking in reptiles.

These physiological disruptions in feeding and metabolism are often associated with reduced growth and condition, although responses vary depending on species and exposure conditions. In fish, reduced growth is frequently accompanied by declines in physiological condition, consistent with impaired energy balance and often associated with disruption of endocrine pathways such as the GH/IGF axis. In amphibians, changes in condition are less consistent and appear to depend on developmental stage, with effects often evident as altered metamorphosis rather than changes in body condition. In reptiles, evidence for effects on condition is limited, and current studies primarily report physical impacts without clear links to systemic energy status.

Environmental factors such as temperature and salinity further modulate these responses, although evidence remains uneven across taxa. In fish, temperature is the most studied factor and often amplifies plastic effects, increasing the severity of impacts on feeding, metabolism, tissue integrity and oxidative stress. Salinity can also influence exposure and toxicity by affecting plastic intake and retention. In amphibians, available data indicate that temperature can modify responses to plastics, although effects are inconsistent. In reptiles, no direct evidence is available on interactions between plastics and environmental factors. There is very limited or no information about other environmental variables such as hypoxia and pH.

A major limitation in the current literature is the high degree of variability among studies. Experimental designs differ in plastic types (polymer composition, size, shape, and whether particles are virgin or environmentally aged), exposure routes (dietary or waterborne exposure), concentrations (reported in different units - e.g., weight/L, number of particles/L, % in feed - and often exceeding environmentally relevant levels), and duration (acute versus chronic). Many studies use pristine, spherical particles under controlled laboratory conditions whereas environmentally relevant plastics are typically weathered, irregularly shaped, and may carry pollutants or pathogenic microbes, which could affect fish health. In addition, studies span a wide range of species with different habitats, physiologies, and feeding strategies, and include multiple life stages (from embryos to adults), experimental conditions and measured endpoints (molecular, physiological, and behavioral). Together, these differences contribute to variability and limit comparability, making broader synthesis and generalizations difficult.

These findings have several practical implications. In aquaculture, plastics present in both water and feed represent a potential risk to animal health and production. MPs have been detected in aquafeeds, which are often derived from small pelagic fish and by-catch, thereby introducing plastic particles into the diets of farmed species (223). This exposure may impair growth and increase physiological stress and disease susceptibility. At the ecosystem level, chronic exposure to plastics may influence population dynamics, reproductive success, and survival, with potential consequences for biodiversity, particularly in sensitive taxa such as amphibians. The bioaccumulation and transfer of plastics and associated contaminants via food webs also raise concerns for food safety and human health.

Several research gaps remain. Current evidence is strongly biased toward fish, with relatively limited information for amphibians and especially reptiles. This taxonomic imbalance, combined with major differences in development (e.g., aquatic larval stages transitioning to terrestrial adults in amphibians, temperature-dependent development in reptiles) and physiology among these groups, makes direct comparisons difficult. Additional studies are needed to determine responses are conserved or taxon-specific. Moreover, many studies rely on short-term exposures and simplified experimental conditions that do not reflect environmental complexity. For example, most research focuses on single polymer types, whereas organisms in natural systems are exposed to heterogeneous mixtures of plastics. Future research should expand on environmentally realistic exposure scenarios, including longer-term studies and the use of mixed plastic types and mixed environmental conditions (e.g., acidification and high temperatures). Greater standardization of experimental approaches would improve comparability across studies. Finally, integrative frameworks linking molecular, physiological, and behavioral responses across species and life stages are needed to better resolve underlying mechanisms.
